# The Critical Contribution of Pseudouridine to mRNA COVID-19 Vaccines

**DOI:** 10.3389/fcell.2021.789427

**Published:** 2021-11-04

**Authors:** Pedro Morais, Hironori Adachi, Yi-Tao Yu

**Affiliations:** ^1^ ProQR Therapeutics, Leiden, Netherlands; ^2^ Department of Biochemistry and Biophysics, Center for RNA Biology, University of Rochester Medical Center, Rochester, NY, United States

**Keywords:** COVID-19, SARS-CoV-2, mRNA, vaccines, RNA modification, pseudouridine, N1-methyl-pseudouridine, lipid nanoparticles

## Abstract

The current COVID-19 pandemic is a massive source of global disruption, having led so far to two hundred and fifty million COVID-19 cases and almost five million deaths worldwide. It was recognized in the beginning that only an effective vaccine could lead to a way out of the pandemic, and therefore the race for the COVID-19 vaccine started immediately, boosted by the availability of the viral sequence data. Two novel vaccine platforms, based on mRNA technology, were developed in 2020 by Pfizer-BioNTech and Moderna Therapeutics (comirnaty® and spikevax®, respectively), and were the first ones presenting efficacies higher than 90%. Both consisted of N1-methyl-pseudouridine-modified mRNA encoding the SARS-COVID-19 Spike protein and were delivered with a lipid nanoparticle (LNP) formulation. Because the delivery problem of ribonucleic acids had been known for decades, the success of LNPs was quickly hailed by many as the unsung hero of COVID-19 mRNA vaccines. However, the clinical trial efficacy results of the Curevac mRNA vaccine (CVnCoV) suggested that the delivery system was not the only key to the success. CVnCoV consisted of an unmodified mRNA (encoding the same spike protein as Moderna and Pfizer-BioNTech’s mRNA vaccines) and was formulated with the same LNP as Pfizer-BioNTech’s vaccine (Acuitas ALC-0315). However, its efficacy was only 48%. This striking difference in efficacy could be attributed to the presence of a critical RNA modification (N1-methyl-pseudouridine) in the Pfizer-BioNTech and Moderna’s mRNA vaccines (but not in CVnCoV). Here we highlight the features of N1-methyl-pseudouridine and its contributions to mRNA vaccines.

## Introduction

When the COVID-19 pandemic struck in early 2020, there was an urgent need to generate COVID-19 vaccines. At that time, the consensus in the medical field was that a safe and effective vaccine would need at least 12–18 months to be developed (Thorp, 2020). Some even argued that such a timeline was highly optimistic since it would have to be tested in animals first during an exploratory and preclinical phase, and then in three different clinical trial phases to determine efficacy and safety ultimately. Finally, a vaccine candidate would need to go through regulatory review, approval, and manufacturing at an unprecedented scale (Kis et al., 2020) with strict quality controls.

To produce effective vaccines and shorten their production time, developing new vaccine strategies/technologies seemed necessary. One of the emerging new technologies, mRNA vaccines (Pascolo, 2004; Probst et al., 2007), drew tremendous attention and provided a great deal of hope. This technology made possible a fast pace of discovery and manufacturing, critical features that could be fully utilized in a biotech and pharmaceutical setting (Jackson et al., 2020).

As opposed to the production of, for example, attenuated or inactivated viruses, the production of mRNA vaccines can take only days or weeks to complete (Pascolo, 2021). It can be accomplished by *in vitro* transcription of mRNA, where virtually any mRNA sequence can be produced from a DNA template (Krieg and Melton, 1984; Melton et al., 1984). Further, an mRNA vaccine would provide the cell with the direct instructions for expressing an immunogenic protein of interest *via* cytoplasmic translation. In fact, it was shown 3 decades ago that an mRNA could be directly delivered, *via* injection, to mouse muscle cells for translation (Wolff et al., 1990). However, as with other nucleic acid-based therapeutic modalities, several delivery hurdles of mRNA therapeutics had delayed the emergence of this technology. For instance, an RNA molecule can be degraded by RNases or entrapped by endosomes before reaching the site of action (Wadhwa et al., 2020). In addition, the negatively charged phosphodiester backbone of an RNA makes it difficult to cross biological membranes (Dowdy, 2017).

The solution to this conundrum was to use a shell of lipid nanoparticles (LNPs) to protect the RNA until it reached the site of action. This is conceptually not very far from what was proposed decades ago, when lipids were tested as vehicles to deliver RNA to mammalian cells (Dimitriadis, 1978; Ostro et al., 1978; Malone et al., 1989). Recently, new generations of LNPs were developed and used to deliver patisiran®, an RNAi-based drug approved in 2018, which generated optimism for RNA therapeutics delivery (Hoy, 2018). Indeed, with the approval of patisiran®, there was a mounting belief that LNPs could become enabling technologies for multiple RNA modalities (Adachi et al., 2021). This was a major accomplishment and a scientific breakthrough, and, in fact, current mRNA vaccines are delivered with LNPs that are prepared by mixing four lipids in the presence of ethanol in very specific conditions (Jeffs et al., 2005; Buschmann et al., 2021). LNPs were also critical for the successful delivery of mRNA vaccines *via* intramuscular injection. It is believed that, while muscle cells are not very efficient in the translation of the mRNA encoding the Spike protein, LNPs ultimately carry their cargo to the lymph nodes and are internalized by dendritic cells. The Spike protein is synthesized in these cells from the mRNA template and displayed to other immune system cells (T and B cells) to trigger the immune response (Ruffell, 2021). Without LNPs formulations, the success of mRNA vaccines would not have been possible.

Aside from the delivery problem discussed above, therapeutic mRNA had at least two additional big challenges: 1) the *in vitro* transcribed (IVT) mRNA would be prone to nuclease degradation when injected into animals, and 2) the IVT mRNA would also lead to innate immunogenicity similar to what would happen when infected by a pathogen (Martinon et al., 1993; Hoerr et al., 2000). The answer to these problems came from a well-known RNA modification, pseudouridine (Ψ), which can be used to replace uridine in the IVT mRNA. It is demonstrated that Ψ can enhance RNA stability and, in the meantime, decrease anti-RNA immune response (Karikó et al., 2008). This Ψ-effect is perhaps associated, at least in part, with the fact that Ψ is a naturally occurring modified nucleotide with unique chemical properties and that Ψ is also highly abundant and naturally widespread in virtually all RNAs of all cells (Song et al., 2020).

Both Pfizer-BioNTech and Moderna Therapeutics COVID-19 spike-encoding mRNA vaccines (both with more than 90% of efficacy against COVID-19 symptoms) contain modified Ψs (Nance and Meier, 2021).

In contrast, another COVID-19 mRNA vaccine candidate (developed by Curevac NV), which is based on an unmodified (Ψ-lacking) mRNA encoding the same COVID-19 spike protein and uses the same LNPs as the Pfizer-BioNTech vaccine does (Buschmann et al., 2021), failed to meet expectations (Baker and Dolgin, 2021). The clinical trial test results ultimately revealed only 48% of efficacy against symptomatic disease (Kremsner et al., 2021) for the unmodified mRNA vaccine, suggesting that modified Ψ and use of LNP technology were both critical success factors for platform validation of mRNA (Dolgin, 2021a). In this mini-review, we will emphasize the main features of this RNA modification and a chemically evolved version of it that contribute to the success of COVID-19 mRNA vaccines and the control of the pandemic.

## Ψ is an Abundant Naturally Occurring Modified Nucleotide Found in Many Types of RNA

**FIGURE 1:**
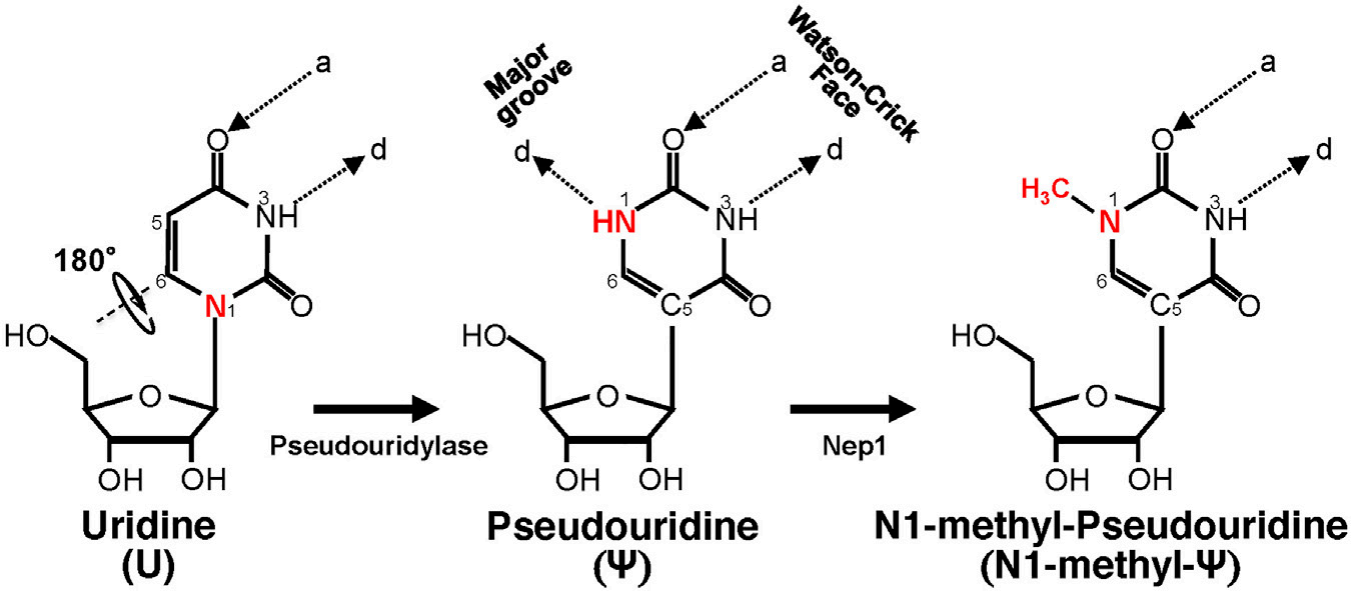
Schematic representation of U-to-Ψ isomerization and additional N1 methylation. Ψ is a rotational isomer of uridine, in which the N-C glycosidic bond is substituted with the C-C bond. The isomerization reaction also creates an extra hydrogen bond donor (-N1H). Ψ can be further methylated at the N1 position by Nep1 (an N1-specific Ψ methyltransferase) to generate N-methyl-Ψ. d, hydrogen bond donor; a, hydrogen bond acceptor.

Ψ was the first modified ribonucleotide discovered 7 decades ago (Cohn and Volkin, 1951; Davis and Allen, 1957), and it has been found in tRNA, rRNA, snRNA, mRNA, and other types of RNA (Carlile et al., 2014; Lovejoy et al., 2014; Schwartz et al., 2014). Ψ is derived from uridine *via* a base-specific isomerization reaction called pseudouridylation (Figure 1), in which the nucleobase rotates 180° around the N3-C6 axis, resulting in the change of nucleobase-sugar bond (from N1-C1′ bond to C5-C1′ bond). The resulting C-C bond allows the nucleobase to rotate more freely (Adachi et al., 2021). In addition, Ψ can provide an extra hydrogen bond donor (in the N1H) in the major groove while keeping the hydrogen bond donor and acceptor (same as in its original uridine) in the Watson-Crick face. While the changes seem subtle (in fact, Ψ can base-pair with adenosine just as uridine does), Ψ can alter RNA structure in a relatively significant way, mainly by improving base-pairing, base stacking, and contributing to making the backbone more rigid (through a network of hydrogen bonding interactions) (Davis, 1995; Charette and Gray, 2000; Newby and Greenbaum, 2001, 2002a, 2002b). As such, RNA pseudouridylation generally stabilizes the RNA. Thus, it is not surprising that the presence of this RNA modification confers distinct biophysical and biochemical properties to the RNA. For example, Ψ favors a C3′-endo conformation in the RNA (Kierzek et al., 2014; Westhof, 2019). Further, it seems that Ψ increases the protection of the RNA against nucleases. A study from Naylor et al. showed that Ψ-containing dinucleotides were more resistant to degradation from snake venom and spleen phosphodiesterases, than the U-containing counterparts (Naylor et al., 1965).

Pseudouridylation can be either catalyzed by stand-alone protein enzymes (pseudouridylases) or by large RNA-Protein complexes called H/ACA box snoRNPs, where the RNA components serve as guides to direct site-specific pseudouridylation (Morais et al., 2021). Since Ψ is highly conserved and known to perform essential functions in the cell, several known diseases are associated with defects in RNA pseudouridylation. Also, because pseudouridylation appears to be irreversible, Ψ is usually excreted from the body. Thus, this RNA modification has drawn attention as a potential biomarker for Alzheimer’s disease and certain types of cancer (Morais et al., 2021).

Ψ can be incorporated into RNA transcripts *via in vitro* transcription, where UTP is replaced by ΨTP (Padilla, 2002; Chen et al., 2010; Pardi et al., 2013). It was reported that Ψ-modified transcripts, coding for four transcription factors (KLF4, c-MYC, OCT4, and SOX2), were successfully used to reprogram human cells to pluripotency with great efficiency (Warren et al., 2010). This landmark study indicated the importance of this RNA modification in mRNA platform technologies.

## Ψ can Trick the Immune System

Upon entering cells, unmodified IVT mRNA becomes intrinsically immunogenic (Weissman et al., 2000). For many years, this challenge slowed down the development of mRNA therapeutics, especially mRNA-replacement strategies. For instance, it has been shown that when treated with unmodified IVT mRNA, dendritic cells promote a T-cell response (Weissman et al., 2000). The activation of Toll-like receptors (TLRs), concretely TLR3 (a member of the TLRs family), that can recognize double-stranded viral RNA, is one of the mechanisms behind this induction of immune response (Karikó et al., 2004). In another work, it was suggested that single-stranded RNA could also induce an immune response in cells. The authors in that work showed that HIV-derived uridine-rich single-stranded RNA could stimulate, *via* recognition by TLR7 and TLR8, dendritic cells to produce cytokines (Heil, 2004). Later, it was further suggested that TLR7 could recognize uracil repeats in close proximity in the RNA (Diebold et al., 2006). To address this problem, Karikó et al. came up with a brilliant solution. They found that incorporating Ψ, as a replacement of uridine, into the IVT mRNA could suppress this immune response mechanism (Karikó et al., 2005). This discovery revealed another critical facet of Ψ and hinted for the first time that RNA modification might be necessary to establish mRNA as a novel therapeutic modality. However, at the time of this finding, some argued that unmodified mRNA immunotherapeutics would be a better approach than modified mRNA since the RNA itself would act as an adjuvant (Ishii and Akira, 2005).

In a follow-up study published in 2008, Karikó et al. proposed that the inclusion of Ψ would be the crucial step for mRNA to mature as a therapeutic tool, both in gene replacement therapies and in mRNA vaccination (Karikó et al., 2008). They confirmed that unmodified mRNA, as compared to Ψ-modified mRNA, was more immunogenic in mice. However, Karikó et al. also suggested that while Ψ-modified mRNA could be preferable for mRNA vaccines, it would eventually require the co-administration of an adjuvant such as lipopolysaccharide or an immunostimulatory oligo. In this regard, it appears that LNPs played this immunoadjuvant role as both carriers and adjuvants for the approved COVID-19 mRNA vaccines (Alfagih et al., 2020).

Another work from the Karikó/Weissman lab suggested that Ψ-modified mRNA could be more resistant to RNase L-mediated degradation (Anderson et al., 2011). This could be achieved by limiting the activation of 2′-5′-oligoadenylate synthetase, an important enzyme in the innate antiviral response that is usually activated by double-stranded RNA. Because RNase L is a 2′-5′-oligoadenylate synthetase-dependent ribonuclease, the ability of pseudouridylated mRNA to limit the activity of 2′-5′-oligoadenylate synthetase could provide an advantage to Ψ-modified mRNA over unmodified mRNA (Anderson et al., 2011).

## Ψ has an Impact on Protein Translation

Because of the impact of Ψ on RNA structure, stability, and chemical properties in general, it is not surprising that this RNA modification also affects the translation of mRNA into protein in eukaryotes. For instance, an early work revealed the unusual decoding events provided by Ψ in the mitochondrial tRNA anticodon. The pseudouridylated anticodon could effectively read alternative codons that would otherwise be poorly recognized during translation in mitochondria if the anticodons were not pseudouridylated (Tomita, 1999). Another study suggested that the increased translatability of Ψ-modified mRNA, which was previously observed (Karikó et al., 2008), was due to the fact that unmodified mRNA is more prone to activate, *via* binding, an RNA-dependent protein kinase (PKR) than Ψ-modified mRNA. This PKR is responsible for the phosphorylation of a translation initiation factor 2-alpha (eIF-2α) and ultimately reduces translation efficiency (Anderson et al., 2010).

Ψ also impacts stop codon decoding. The Yu lab showed that nonsense mutations, which create premature termination codons (PTCs), could be suppressed by site-specific pseudouridylation of the uridine of PTCs (UAG, UGA, and UAA) directed by artificial box H/ACA guide RNAs (Karijolich and Yu, 2011; Morais et al., 2020). The identity of the amino acids incorporated in the pseudouridylated PTCs was determined in yeast by immunoprecipitation and mass spectrometry: predominantly phenylalanine/tyrosine at the ΨGA codons and threonine/serine at the ΨAA and ΨAG codons. It was later found that this novel recoding mechanism could happen due to an unusual codon-anticodon base-pairing scheme at the ribosomal decoding center (Fernández et al., 2013).

More recently, it was reported that Ψ is also capable of modulating translatability or sense codon decoding (Eyler et al., 2019). Using either an *Escherichia coli* translation system or human cells (human embryonic kidney cells), the authors demonstrated that Ψ could alter, to a small extent, how ribosomes or codons interact with cognate and near-cognate tRNAs, leading to amino acid substitution. It was suggested that this amino acid substitution mechanism could be a valuable source for adaptation under stress conditions, such as oxidative and temperature stresses.

## N1-Methylated Ψ Behaves Better Than Ψ

Since the finding that Ψ-modification could enable mRNA to resist intrinsic immune responses (Karikó et al., 2005), a search was carried out for Ψ-derivatives that could have improved properties. The amine group (NH) at the N1 position, which provides an extra hydrogen bond donor (created after pseudouridylation) (Figure 1), drew particular attention. One N1-modified Ψ-derivative is N1-methyl-Ψ, a naturally occurring modification found in 18S rRNA (Brand et al., 1978) and tRNA in many organisms (Boccaletto et al., 2018). This N1-methylation is catalyzed by N1-specific Ψ methyltransferase Nep1 found in archaea and eukaryotes (Wurm et al., 2010) (Figure 1). Potentially N1-methyl-Ψ could be more widespread than reported in human RNA, given that the current standard Ψ-detection (-seq) methods, which rely on the use of CMC-modification followed by primer extension (Morais et al., 2021), may not be able to distinguish N1-methyl-Ψ from Ψ (Svitkin et al., 2017). Possibly, therefore, some Ψs thus identified so far (Schwartz et al., 2014) could actually be N1-methylated Ψs.

In order to understand the biological functions of N1-methyl-Ψ, Parr et al. performed biophysical studies where this modification was compared with Ψ and uridine. They measured the melting temperature of complementary synthetic RNA duplexes in which some uridines were replaced by Ψ or N1-methyl-Ψ (Parr et al., 2020). Both the Ψ- and N1-methyl-Ψ-modified duplexes had higher (and similar) T_m_-values than uridine-control duplexes, indicating higher stability provided by increased base pairing and stacking as suggested in previous studies performed with Ψ (Westhof, 2019). However, Ψ contains an extra hydrogen bond donor group (N1H) that contributes to a universal base character, i.e., it can not only pair A but also wobble base-pair with G, U, or C in the context of a duplex (Kierzek et al., 2014). On the other hand, N1-methyl-Ψ has a methyl group instead in the N1-position (Figure 1), thus eliminating the extra hydrogen bond donor. Consequently, N1-methyl-Ψ can only use its Watson-Crick face to base-pair with another nucleoside, thus preventing it from wobble-pairing with other nucleotides (G, U, and C). Nonetheless, Ψ and N1-methyl Ψ still share a critical common feature, the C5-C1′ bond, which enables rotation between the nucleobase and the sugar moieties and probably contributes to improving the base-pairing, base-stacking, and duplex stability (Westhof, 2019). It is conceivable that N1-methylated Ψ, which has a higher affinity for pairing with A (similar to Ψ) and is less likely to activate PKR, would be more efficient for translation when compared to uridine. On the other hand, N1-methyl-Ψ remains faithful in coding (more like uridine than Ψ does in pairing) during translation. Finally, N1-methyl-Ψ, which is structurally similar to Ψ, would probably also enable mRNA to evade the immune response.

Indeed, it has been reported that N1-methyl-Ψ diminished the activity of innate immune sensors (Andries et al., 2015) and that N1-methyl-Ψ performed nicely (and even better than Ψ) in improving the translational capacity and reducing cytotoxicity of modified mRNA when tested in several human cell lines, primary human cells, and in animals (intradermal and intramuscular injection in mice) (Andries et al., 2015). Some of the findings were later corroborated by scientists from Moderna Therapeutics (Nelson et al., 2020). Furthermore, another study by Svitkin et al. confirmed the effect of N1-methyl-Ψ on innate immune sensors and demonstrated that N1-methyl-Ψ increased ribosome pausing and thus change the dynamics of modified mRNA translation by increasing the recruitment or loading of ribosomes (Svitkin et al., 2017). Due to its effectiveness, N1-methyl-Ψ (alone or in conjunction with 5-methylcytidine) was thus proposed to be a new benchmark in RNA modifications for mRNA therapeutics (Andries et al., 2015).

## N1-Methyl-Ψ is Used in Covid-19 mRNA Vaccines

In 2017 during the development of mRNA vaccine against Zika virus, N1-methyl-Ψ was used and incorporated into two similar mRNA vaccines encoding Zika virus surface proteins. The modified mRNA, encapsulated in LNPs, was designed and then tested to protect against the Zika virus in human cells, mice, and non-human primates (Pardi et al., 2017; Richner et al., 2017). In the following year, further success was obtained with N1-methyl-Ψ-modified mRNA vaccines against HIV-1, Zika, and influenza virus, achieving a sustained antibody response in a preclinical setting (Pardi et al., 2018). A similar example was presented against the Ebola virus in guinea pigs (Meyer et al., 2018). These studies further emphasized the importance of N1-methyl-Ψ for the mRNA vaccine platform technology, as it could provide a reliable way of achieving the sustained and speedy synthesis of the antigenic protein to trigger the desired immune response in a safe manner.

In 2020, Pfizer-BioNTech added N1-methyl-Ψ to their COVID-19 mRNA vaccine candidate (comirnaty® or BNT162b2) coding for the full-length transmembrane S protein “spike.” The full sequence of this mRNA vaccine includes the 5′UTR, the coding sequence of the spike protein with two contiguous stop codons, and the 3′UTR (Nance and Meier, 2021). N1-methyl-Ψ was substituted for all uridines throughout the mRNA sequence, including the uridines in the two stop codons. In addition, two amino acid mutations, K986P and V987P (lysine 986 and valine 987 were both changed to proline), were also introduced. These mutations help generate the pre-fusion conformation of the spike protein that is more optimal as an antigen since it more resembles the actual viral protein with which antibodies will interact (Pallesen et al., 2017; Wrapp et al., 2020). In an earlier study of MERS-CoV infection, it was found that the two prolines would stabilize the pre-fusion conformation of the MERS-CoV spike antigen (Pallesen et al., 2017). Antibodies generated against this conformation would block the fusion of the virus and the host protein (CD26), thus offering an ideal solution for MERS-disease vaccine development. This knowledge was incorporated into the development of COVID-19 mRNA vaccine (Pfizer-BioNTech and Moderna) and non-mRNA vaccines as well (J&J and Novavax vaccines) (Kyriakidis et al., 2021).

Massive *in vitro* transcription produced a huge amount of N1-methyl-Ψ-modified SARS CoV-2 (COVID-19) spike mRNA. This vaccine was the first mRNA vaccine fully approved against COVID-19 after showing a good safety profile and 95% protection against disease following a two-dose regimen (intramuscular injection) (Polack et al., 2020; Mullard, 2021).

The Moderna Therapeutics COVID-19 vaccine (spikevax®, or mRNA-1273), also coding for pre-fusion conformation of the spike protein (Corbett et al., 2020), was the second mRNA vaccine to get EAU (emergency approval use) for COVID-19. Spikevax® was also prepared by totally replacing uridines with N1-methyl-Ψ through *in vitro* transcription (Corbett et al., 2020). The spike protein-coding sequence ends with three N1-methyl-pseudouridylated stop codons and is flanked by a 5′UTR and a 3′UTR. This vaccine was shown to prevent COVID-19 disease, including severe illness, with an efficacy of 94% (Baden et al., 2021).

It is worth noting that although the mRNA of both approved vaccines is fully modified (Us are completely substituted with N1-methyl-Ψs), it likely has high coding fidelity, given that N1-methyl-Ψ pairs only with A (unlike Ψ, which can, to some extent, wobble pair with different nucleosides). In addition, two and three contiguous stop codons are placed in the Pfizer and Moderna mRNAs, respectively. Such arrangements ensure that no read-through of modified stop codons will occur (even though a single Ψ-stop codon would allow, to some extent, read-through) (Karijolich and Yu, 2011; Fernández et al., 2013). Also, N1-methyl-Ψ increases translation efficiency, which enables relatively low doses.

## Modified vs. Unmodified COVID-19 mRNA Vaccines Lead to Different Outcomes

The intrinsic immunogenicity of non-modified mRNA was once considered a potential advantage for its use in vaccines (Ishii and Akira, 2005) as it would encode the antigen and concomitantly serve as an adjuvant while permitting a low dose. In fact, the unmodified COVID-19 mRNA vaccine candidate in late-stage clinical trials (CVnCoV, developed by Curevac) had a maximum dose of 12 µg. However, the recent CVnCoV vaccine clinical trial results showed only 48% of efficacy against any severity of the disease, (Kremsner et al., 2021).

In light of such results, some argued that this could be due to a dose that was too low to elicit a robust immune response against the disease [higher doses of the unmodified mRNA vaccine appear to be intolerable to patients (Dolgin, 2021a; Cohen, 2021)]. Consistent with this argument, Pfizer-BioNTech and Moderna’s mRNA vaccines, which exhibit ∼95% high protection rate against COVID-19, come with a much higher dose, by comparison, 30 and 100 µg of modified mRNA each shot, respectively (Pascolo, 2021). Although lower doses (50 and 25 µg) of Moderna’s modified mRNA-1273 can still elicit a significant immune response (Chu et al., 2021; Mateus et al., 2021), they remain much higher than the doses of CVnCoV unmodified mRNA vaccine. Interestingly, however, Pfizer-BioNTech just announced that their comirnaty® vaccine, administered with two shots of 10 µg each, is safe and effective in children 5–11 years old (Pfizer, 2021). There is some speculation surrounding the possibility that, although designed for children, this dose is comparable to the dose of the CVnCoV unmodified mRNA vaccine; thus, it would not be the low dose that made the unmodified mRNA vaccine relatively ineffective. This hypothesis warrants further study.

**FIGURE 2:**
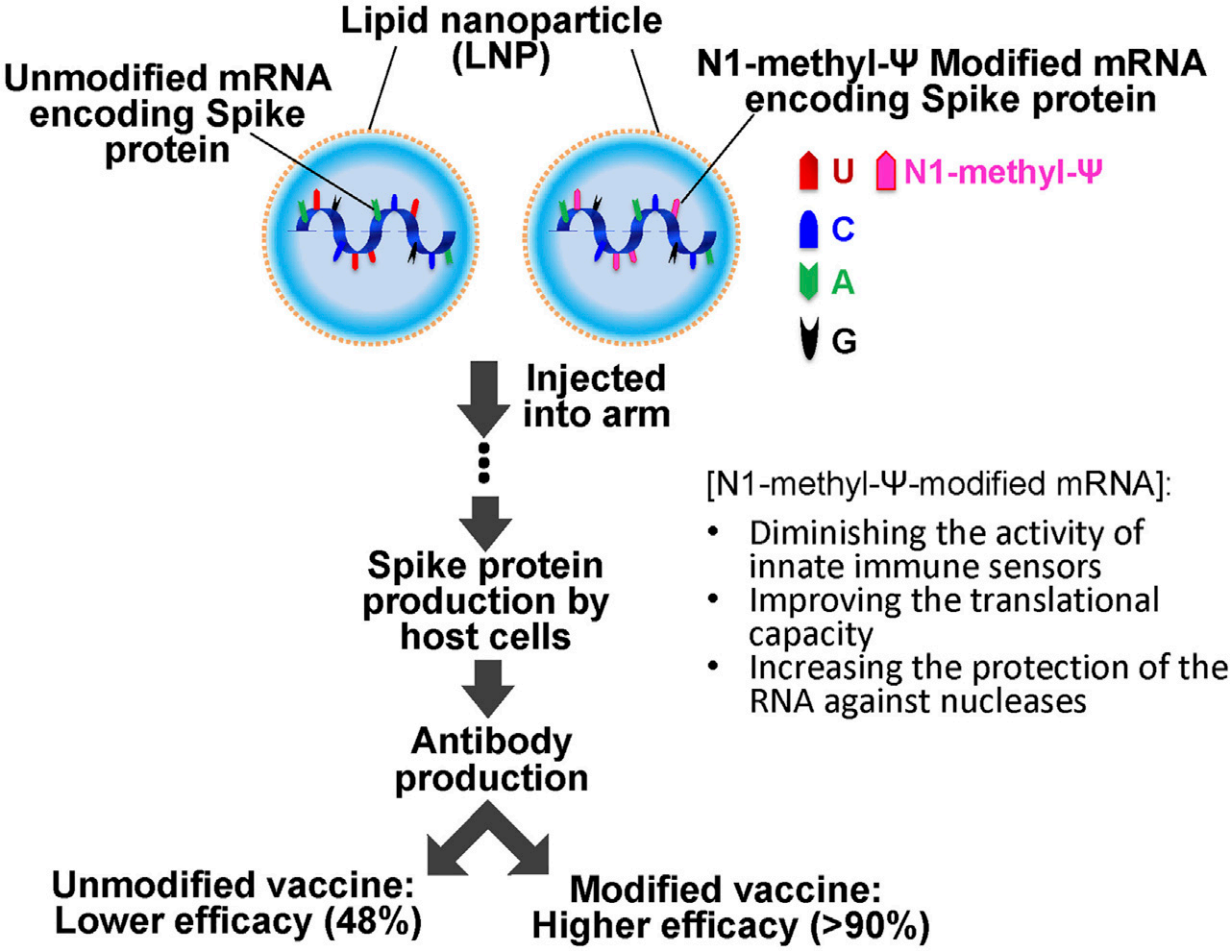
Schematics of SARS-COVID 19 mRNA vaccination. The vaccine consists of unmodified or N-methyl-Ψ-modified mRNA (encoding the SARS-COVID-19 spike protein) and lipid nanoparticles (LNPs). It is injected into the muscle of the upper arm to create an immune response. N-methyl-Ψ-modified mRNA exhibits higher efficacy (more than 90% of efficacy against COVID-19 symptoms) as compared to the unmodified mRNA vaccines (lower than 50%).

It should also be pointed out that the CVnCoV unmodified mRNA vaccine also used an LNP formulation, namely Acuitas ALC-0315, a delivery system identical to that used in the Pfizer-BioNTech modified mRNA vaccine (Buschmann et al., 2021). While Curevac attributed the lower efficacy of CVnCoV to the large number of variants circulating during the clinical trials, this claim has been challenged by the high protection of the Pfizer–BioNTech mRNA vaccine against the alpha, beta and delta variants (92, 75, and 83% respectively) (Abu-Raddad et al., 2021; Sheikh et al., 2021). Given these experimental and clinical trial results, one could argue that RNA modifications are perhaps critical contributors to the success of the mRNA vaccine platform technology (Dolgin, 2021a) (Figure 2).

The second-generation of Curevac’s COVID-19 vaccine (CV2CoV), currently in preclinical development (Roth et al., 2021), is still a non-chemically modified mRNA, which encodes the full-length spike protein and is encapsulated with LNPs. Compared to the first generation of Curevac COVID-19 unmodified mRNA vaccine, the second-generation unmodified mRNA vaccine consists of coding and non-coding (5′ and 3 UTRs) sequences that have been further engineered to increase translation efficiency and antigen protein production. In a study published before the pandemic, Curevac (and Acuitas) scientists presented data suggesting that the use of unmodified mRNA could be compensated by heavily engineering the sequence of the mRNA to enhance protein expression (erythropoietin) in mice and large animals (Thess et al., 2015). They optimized the codons in the open reading frame and thus improved the stability and translation of the unmodified transcript. Of note is that both Pfizer-BioNTech and Moderna mRNA vaccines are already codon/sequence optimized.

It is possible that the second generation of Curevac’s COVID-19 mRNA vaccine, CV2CoV, which has already shown increased levels of neutralizing antibodies in rats (Roth et al., 2021), will enhance the safety and protection profile. The clinical trial results are expected to come in 2022. In the meantime, another unmodified mRNA vaccine (ARCoV), developed by Walvax Biotechnology and Suzhou Abogen Biosciences, is currently in clinical development (Dolgin, 2021b). In addition, Sanofi, a French pharmaceutical company, which recently acquired an unmodified mRNA technology platform from Translate Bio, now a Sanofi company, recently announced the discontinuation of their phase ½ clinical trials of their Sanofi-Translate Bio unmodified COVID-19 mRNA vaccine to focus their efforts instead in their influenza vaccine which is based on modified RNA (Sanofi, 2021). Curevac has also recently withdrawn CVnCoV from the regulatory approval process to focus their efforts instead on their second-generation CV2CoV vaccine clinical development. Moreover, the company stated that it will accelerate the development of modified mRNA vaccine constructs, in collaboration with GlaxoSmithKline, a pharmaceutical company (Curevac, 2021).

Unmodified mRNA is being used in non-COVID-19 clinical trials, particularly for developing new cancer treatments. It has been suggested that the challenge associated with the activation of an immune response against cancer cells could be better surmounted with the use of unmodified mRNA (with its stronger adjuvant activity) coding for proteins usually present in cancer cells but not in healthy cells, in order to turn a cold tumor into a hot tumor more effectively (Ruffell, 2021). In fact, BioNTech just announced the use of unmodified mRNA encapsulated in a lipoplex delivery formulation, following this concept, for treatment of colorectal cancer patients in phase two trials (BioNTech, 2021).

Regardless, it is clear that RNA modifications, such as Ψ and later N1-methyl-Ψ, have already made a tremendous and timely contribution to generating highly effective (+90%) COVID-19 mRNA vaccines. Pfizer-BioNTech’s mRNA vaccine went from first-in-human trials to emergency use authorization in just 8 months (Dolgin, 2021b).

While mutations in COVID-19 are leading to new variants that pose increasing challenges and that require further study of the efficacy of currently approved vaccines, there is no doubt that the developments in biology and chemistry of the most common RNA modification (Ψ) during the last 2 decades have turned out to be game-changing in defining how to end this pandemic.
